# Proximal femoral tumor resection followed by joint prosthesis replacement: a systematic review and meta-analysis

**DOI:** 10.1186/s12891-023-06913-w

**Published:** 2023-10-02

**Authors:** Bo Li, Yongzhi Yu, Yun Bao, Jianmin Song

**Affiliations:** 1 Department of Musculoskeletal Tumor, Gansu Provincial Hospital, Lanzhou, China; 2 Institute of Clinical Research and Evidence Based Medicine, Gansu Provincial Hospital, Lanzhou, China

**Keywords:** Proximal femoral tumor, Prosthetic reconstruction, limb salvage, Meta-analysis

## Abstract

**Background:**

This study aimed to determine the prognostic outcome of hip joint replacement after resection of proximal femoral tumors by reviewing original studies.

**Methods:**

Two researchers independently searched PubMed, Embase, Cochrane Library, and Web of Science databases from inception to July 17, 2022. Then, the literature was screened by inclusion criteria. The basic information, primary outcomes, and secondary outcomes were extracted for weighted combined analysis. The quality of the included literature was evaluated using the Newcastle–Ottawa scale.

**Results:**

Twenty-four retrospective cohort studies comprising 2081 patients were included. The limb salvage rate was 98%. The survival rates at 1, 2, 3, 4, and 5 years were 80, 72, 65, 64, and 55% for patients with primary tumors and the rate at 1, 2, 3, 4, and 5 years were 44, 25, 17, 14, and 11% for patients with bone metastases, respectively.

**Conclusion:**

As chemotherapy and radiotherapy treatment progressed, joint reconstruction after proximal femoral tumor resection improved patients' function and quality of life.

**Supplementary Information:**

The online version contains supplementary material available at 10.1186/s12891-023-06913-w.

## Background

Primary and secondary bone tumors can occur in bone and cartilage tissues [1]. Primary bone tumors occur in children and adolescents and are an important contributor to death and disability in this age group [2]. Secondary bone tumors, i.e., bone metastases, are 30–40 times more common than primary ones and are characterized by higher prevalence and poor quality of life [3]. Except for the spine, the proximal femur is the most frequent site of bone metastases (approximately 10%) and is the most commonly affected long bone [4]. The risk of pathologic fracture of the proximal femur metastases is high due to weight bearing and biomechanical conditions [5].

One of the most important objectives in treating proximal femoral tumors is to reduce pain to provide a better quality of life for the patient. Limb-preserving surgery has gradually become the main surgical modality for bone tumors [6]. With the advancement of relevant, comprehensive treatments, the goal of treatment for bone tumors requires improving patient survival and preserving good limb function [7]. Metal tumor-based artificial joints are currently the first choice for functional reconstruction after limb preservation surgery due to the advantages of immediate postoperative restoration of affected limb function, early mobility, and long-term functional satisfaction [8].

The preferred reconstruction method for patients with proximal femoral tumors is prosthetic replacement after tumor resection. Due to the low incidence and limited follow-up time, reports of clinical outcomes, survival, prosthetic survival, and function of patients after surgery vary widely in original studies. A systematic evaluation meta-analysis published by Thambapillary et al. [9] in 2013 reported a limb preservation rate of over 90%, a 5-year prosthetic survival rate of 84%, and an overall revision rate of 11% without addressing patient survival. In contrast, the systematic evaluation published by Brown et al. [10] in 2018 only qualitatively described the relevant data. Based on the publication of several relevant original studies in recent years, this study intends to update and supplement the analysis of clinical outcomes, complications, survival, prosthetic survival, and function of metal prosthesis replacement after proximal femoral tumor resection and subgroup analysis was according to follow-up time, prosthesis type, and tumor type to bring reference for clinical decision-making.

## Material and methods

### Literature search

This systematic review was reported according to PRISMA (Preferred Reporting Items for Systematic Reviews and Meta-analysis) guidelines. Two investigators independently conducted database searches, and arguments were resolved through discussion. PubMed, Embase, Cochrane Library, and Web of Science databases were searched from inception to July 17, 2022. The search strategy was "Neoplasms" AND "Arthroplasty, Replacement, Hip" and was limited to English-language papers.

### Eligibility criteria

The inclusion criteria were: 1) patients diagnosed with a proximal femoral tumor; 2) hip replacement after tumor resection; 3) cohort study. The exclusion criteria were: 1) number < 10; 2) conference abstracts, and reviews; 3) revision surgery; 4) unavailability of the full text.

### Literature screening and data extraction

Two investigators independently screened the literature and extracted data. After literature de-duplication, irrelevant literature was excluded by reading the title and abstract, and the full text of relevant literature was further analyzed. Data were extracted based on a pre-designed table, including authors, year of publication, country, total number, gender, age, and follow-up time. The primary outcomes included overall patient survival and limb preservation rate. The secondary outcomes included limb salvage rate (the proportion of patients who avoided amputation at end of follow-up period), prosthesis survival, revision rate, hemi to total hip conversion rate, tumor status, complications, and function based on the Musculoskeletal Tumor Society, 1993, MSTS.

### Risk of bias assessment

The quality of the included literature was assessed using the Newcastle–Ottawa scale (NOS). The following characteristics were assessed: representativeness of the exposed cohort, selection of the non-exposed cohort, ascertainment of exposure, the demonstration that the outcome of interest was not present at the start of the study, comparability of cohorts based on the design or analysis, or outcome assessment, whether the follow-up was long enough for outcomes to occur, and adequacy of follow-up of cohorts.

### Data synthesis and statistical analysis

Data were analyzed using R software (v. 6.0). Individual rates were combined using double inverse sine transformation [11], and the rate and 95% confidence intervals (CIs) were calculated. Cochran-Q test for heterogeneity and I^2^ evaluated the magnitude of heterogeneity. An I^2^ > 50% was considered high heterogeneity, and a random effects model was used; for I^2^ ≤ 50%, a fixed effects model was used. The survival data were captured from Kaplan–Meier (K-M) curves using Engauge Digitizer software. The subgroup analysis was performed according to follow-up time, prosthesis type, and tumor type. The publication bias was assessed using Egger's test, and the sensitivity analysis was performed by excluding individual studies on a case-by-case basis.

## Results

### Literature search results

**Fig. 1:**
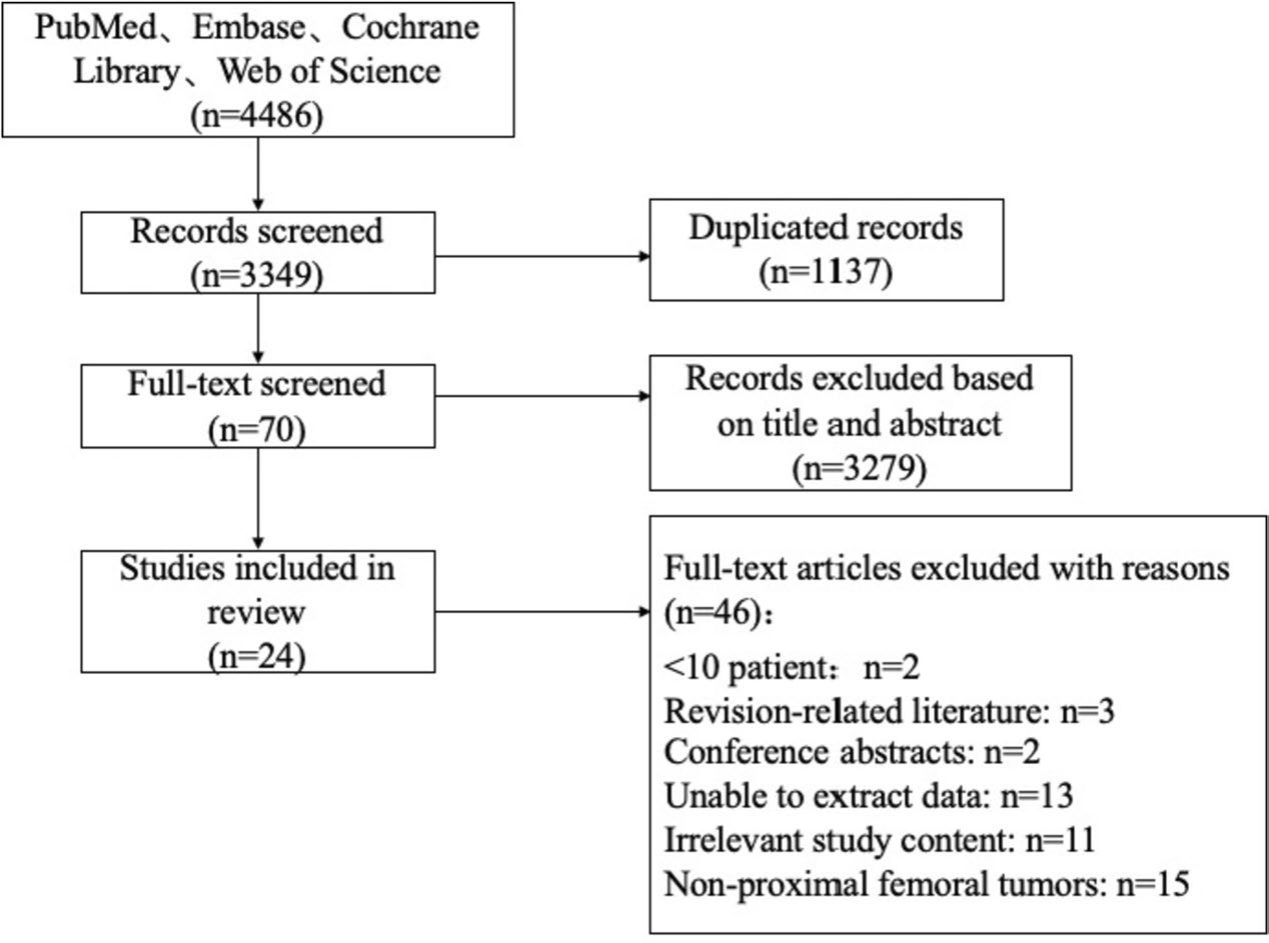
Flow diagram of the study selection process

A total of 4486 studies were searched. After excluding duplicates (1137), the title and abstract of 3349 studies were analyzed, and 70 studies were selected for full-text screening. Two studies with < 10 patients, three revision-related reports, two conference abstracts, 13 with unextractable data, 11 with irrelevant study content, and 15 non-proximal femoral tumors were excluded. Finally, 24 studies were included for analysis. The selection process is shown in Fig. 1.

### Studies description

The 24 included papers were retrospective cohort studies (Table 1) comprising 2081 patients, with the number of males ranging from 4–236 in each study and the number of females ranging from 8–468. The age at surgery ranged from 27.8–66.1 years, and the follow-up period ranged from 3–216 months. The year of publication for each study ranged from 1998–2020. The countries with the most published studies were the United States (12), China (3), and the United Kingdom (2).

**Table 1:** Basic characteristics of included studies

Study	Publication Year	Country	Study type	Study time	Number of patients	Male/Female	Surgery age	Follow up time (year)	NOS
Atalay,et al [12]	2020	Turkey	R cohort	NA	12	4/8	66.0(19–84)	3	8
Bernthal, et al [13]	2010	USA	R cohort	1982.12–2008.12	86	40/46	44.5(10–83)	64.4(3–291)	7
Bischel, et al [14]	2020	Germany	R cohort	1997–2000	45	19/26	58.7(34–85)	16.4(0.6–74.7)	7
Chandrasekar,et al [15]	2009	UK	R cohort	2001–2006	100	52/48	56.3(16–84)	24.6(0–60)	6
Farid, et al [16]	2006	USA	R cohort	1974–2002	52	24/28	P: 32(16–87) M:52(34–72)	Min:24	6
Finstein, et al [17]	2007	USA	R cohort	1981–2003	62	30/32	49(10–83)	60(1–259)	6
Hobusch, et al [18]	2017	Austria	R cohort	1979.1–2010.6	16	5/11	26(11–49)	216 (60–324)	7
Houdek, et al [19]	2016	USA	R cohort	1969–2014	204	112/92	59(11–88)	84(24–264)	7
Houdek, et al [20]	2019	USA	R cohort	2000–2015	148	81/67	57(11–88)	59(37–91)	8
Jacofsky,et al [21]	2004	USA	R cohort	1980–2000	42	22/20	63(20–84)	59(0.5–240)	7
Johnson, et al [22]	2019	USA	R cohort	2000–2016	26	15/11	63 ± 11	72 ± 48	6
Liu, et al [23]	2018	China	R cohort	2010.1–2015.1	32	15/17	27.8 (36–84)	66(32–84)	7
Manoso, et al [24]	2007	USA	R cohort	1994–2000	13	5/8	62(46–77)	NA	6
Meynard,et al [25]	2020	France	R cohort	2001.1–2017.12	161	NA	NA	NA	8
Nakashima, et al [26]	2010	Japan	R cohort	1993–2006	40	22/18	63.4(31–81)	17(1–92.5)	7
Nooh, et al [27]	2020	Canada	R cohort	2000–2019	47	59%	59(15–89)	44(1–228)	6
Peterson, et al [28]	2017	USA	R cohort	2012–2015	21	11/10	Male:43–87 Female:8–85	Male:11(1–27) Female:18(12–27)	7
Potter, et al [29]	2009	USA	R cohort	1993–2003	59	33/26	58(10–88)	55.4(24–152)	7
Sokolovski, et al [30]	2006	Russia	R cohort	1994–2004	44	23/21	39(13–80)	12–120	6
Varady, et al [31]	2019	USA	R cohort	2007–2017	704	236/468	Hemi:71.4 ± 1.3 Total:66.1 ± 1.5	NA	7
Wu, et al [32]	2016	China	R cohort	2005.1–2014.6	28	19/9	Total:32.3 ± 15.9 Hemi: 48.3 ± 21.3	30.5 (6–108)	7
Yu, et al [33]	2018	China	R cohort	2005.1–2014.12	57	34/23	62.5	NA	7
Clarke, et al [34]	1998	USA	R cohort	1984.1–1995.12	28	11/17	62.9(29–81)	24.7(2–91)	6
Kabukcuoglu, et al [35]	1999	UK	R cohort	1972–1992	54	31/23	40.2(15–76)	108(5–288)	6

*P* Proximal tumor, *M* Bone metastases, *R* Retrospective

### Methodological quality of included studies

The results of the NOS evaluation form showed that three of the 24 articles scored 8, 12 scored 7, and nine scored 6. There was no literature with a high risk of bias.

### Intraoperative bleeding, operative time, hospital days, and postoperative function

Four studies (123 patients) reported intraoperative bleeding in patients with a bleeding volume of 630 mL (95% CI: 501.53–759.28, I^2^ = 94%). Five studies (827 patients) reported an operative time of 123.85 min (95% CI: 110.30–137.39, I^2^ = 100%). Four (154 patients) reported the number of hospital days (10.47 d, 95% CI: 8.64–12.30, I^2^ = 99%). Nine studies (265 patients) measured the postoperative patient function using the MSTS 93 scale, with a combined score of 22.5 (95% CI: 20.43–24.56, I^2^ = 98%) (Table 2).

**Table 2:** Bleeding volume, operative time and hospital days

	Study	Effect size	95%CI	I^2^(%)
bleeding volume (ml)	4	630.41	501.53–759.28	94
Operative time (min)	6	123.85	110.30–137.39	100
hospital days (day)	4	10.47	8.64–12.30	99
patient function (MSTS)	9	22.5	20.43–24.56	98

### Survival rate of patients

Patient survival rates at 1, 2, 3, 4, and 5 years were 69% (95% CI: 56–82%), 53% (95% CI: 37–69%), 46% (95% CI: 28–64%), 42% (95% CI: 22–62%), and 36% (95% CI: 24–48%), respectively (Additional file 2: Appendix Table 1). Patients with primary tumors, custom-made prostheses, and total hip replacements have a higher survival rate than those with metastases, modular-made prostheses, and hemi-hip replacements. The subgroup analysis according to tumor type showed that the survival rates at 1, 2, 3, 4, and 5 years were 80, 72, 65, 64, and 55% for patients with primary tumors and rate at 1, 2, 3, 4, and 5 years were 44, 25, 17, 14, and 11% for patients with bone metastases, respectively. The subgroup analysis according to the type of prosthesis showed that the 1, 2, 3, 4, and 5-year survival rates were 93, 80, 70, 67, and 60% for patients with custom-made prostheses, 66, 48, 38, 37, and 32% for patients with modular-made prostheses. The 1, 2, 3, 4, and 5-year survival rates were 73, 56, 51, 48, and 47% for patients with hemi-hip replacements and 93, 82, 74, 69, and 57% for patients with total hip replacements, respectively (Table 3).

**Table 3:** Subgroup analysis of patient survival rates

Follow-up time	Subgroup	Number of Studies	Number of patients	rates	95%CI	I^2^(%)
1^st^ year	Primary	6	355	80%	72–87	0%
	metastasis	3	112	44%	35–54	71%
	Custom	2	72	93%	87–99	0%
	Modular	3	252	66%	57–75	52%
	Hemi hip	4	281	73%	65–82	59%
	Total hip	2	60	93%	87–100	0%
2^nd^ year	Primary	2	87	72%	62–81	0%
	metastasis	5	290	25%	16–35	70%
	Custom	2	72	80%	71–89	36%
	Modular	3	252	48%	34–63	80%
	Hemi hip	4	281	56%	50–61	0%
	Total hip	2	60	82%	73–92	0%
3^rd^ year	Primary	2	87	65%	55–75	0%
	metastasis	5	290	17%	11–23	51%
	Custom	2	72	70%	54–86	54%
	Modular	3	252	38%	13–62	94%
	Hemi hip	4	281	51%	45–57	0%
	Total hip	2	60	74%	63–85	15%
4^th^ year	Primary	2	87	64%	54–74	22%
	metastasis	5	290	14%	5–24	87%
	Custom	2	72	67%	56–78	0%
	Modular	3	252	37%	13–61	94%
	Hemi hip	4	281	48%	42–53	0%
	Total hip	2	60	69%	57–80	0%
5^th^ year	Primary	3	112	55%	46–65	0%
	metastasis	6	355	11%	5–18	81%
	Custom	2	72	60%	49–71	45%
	Modular	3	252	32%	8–57	95%
	Hemi hip	5	364	47%	42–52	31%
	Total hip	2	60	57%	36–78	58%

### Survival rate of prosthesis

The survival rates for prostheses at 1, 2, 3, 4, and 10 years were 98% (95% CI: 96–100%), 97% (95% CI: 95–100%), 89% (95% CI: 83–96%), 88% (95% CI: 80–95%), and 73% (95% CI: 58–89%) (Additional file 2: Appendix Table 1). Prosthesis survival did not differ between tumour types or prosthesis types. The subgroup analysis according to tumor type showed that the 1 and 2-year prosthesis survival rates were 100% for patients with primary tumors and bone metastases. The subgroup analysis according to prosthesis type showed that the 1- and 2-year survival rates were 100 and 95% for custom-made prostheses, 100% for modular-made prostheses, 94 and 93% for hemi-hip replacement, and 100 and 95% for total hip replacement, respectively (Table 4).

**Table 4:** Subgroup analysis of prosthetic survival

Follow-up time	Subgroup	Number of studies	Number of patients	rates	95%CI	I^2^(%)
1^st^ year	Primary	3	59	99%	95–100	41%
	metastasis	3	128	100%	98–100	0%
	Custom	5	344	97%	95–100	60%
	Modular	4	300	99%	97–100	65%
	Hemi hip	1	44	100%	97–100	NA
	Total hip	2	97	100%	98–100	0%
2nd year	Primary	2	148	94%	88–100	53%
	metastasis	1	44	100%	97–100	NA
	Custom	1	34	100%	90–100	NA
	Modular	2	63	100%	97–100	0
	Hemi hip	4	244	96%	92–100	65
	Total hip	3	200	96%	91–100	75

### Prosthesis revision rate

**Fig. 2:**
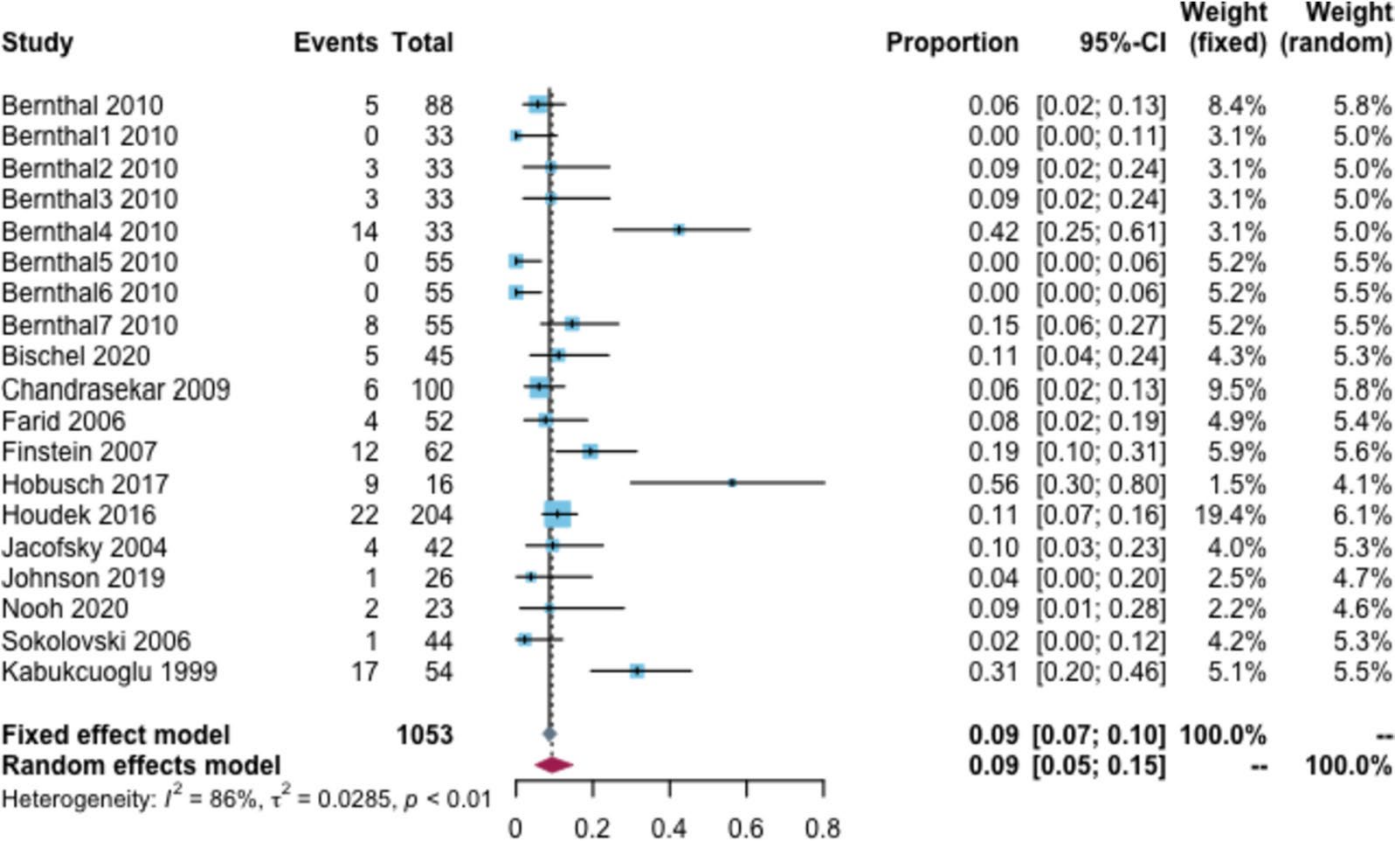
Revision rate of prosthetic

The overall prosthesis revision rate was 9% (95% CI: 5–15%, I^2^ = 86%) (Fig. 2). The subgroup analysis based on follow-up time showed that the revision rates were 2, 8, 21, and 47% at 2, 5, 10, and 20 years, respectively. Moreover, the subgroup analysis based on prosthesis type showed that the revision rate was 11% for custom-made prostheses and 8% for modular-made prostheses (Additional file 2: Appendix Table 2). Additionally, three studies described the causes of revision, with the main causes being aseptic loosening and infection.

The rate of hemi to total hip conversion was 3% (95% CI: 1–6%, I^2^ = 74%) (Additional file 2: Appendix Fig. 1). The subgroup analysis based on follow-up time showed revision rates of 2 and 5% at 2–5 and 6–10 years of follow-up, respectively. Besides, the prosthesis type subgroup analysis showed a revision rate of 3% for custom-made prostheses (Additional file 2: Appendix Table 3).

### Limb salvage rate

**Fig. 3:**
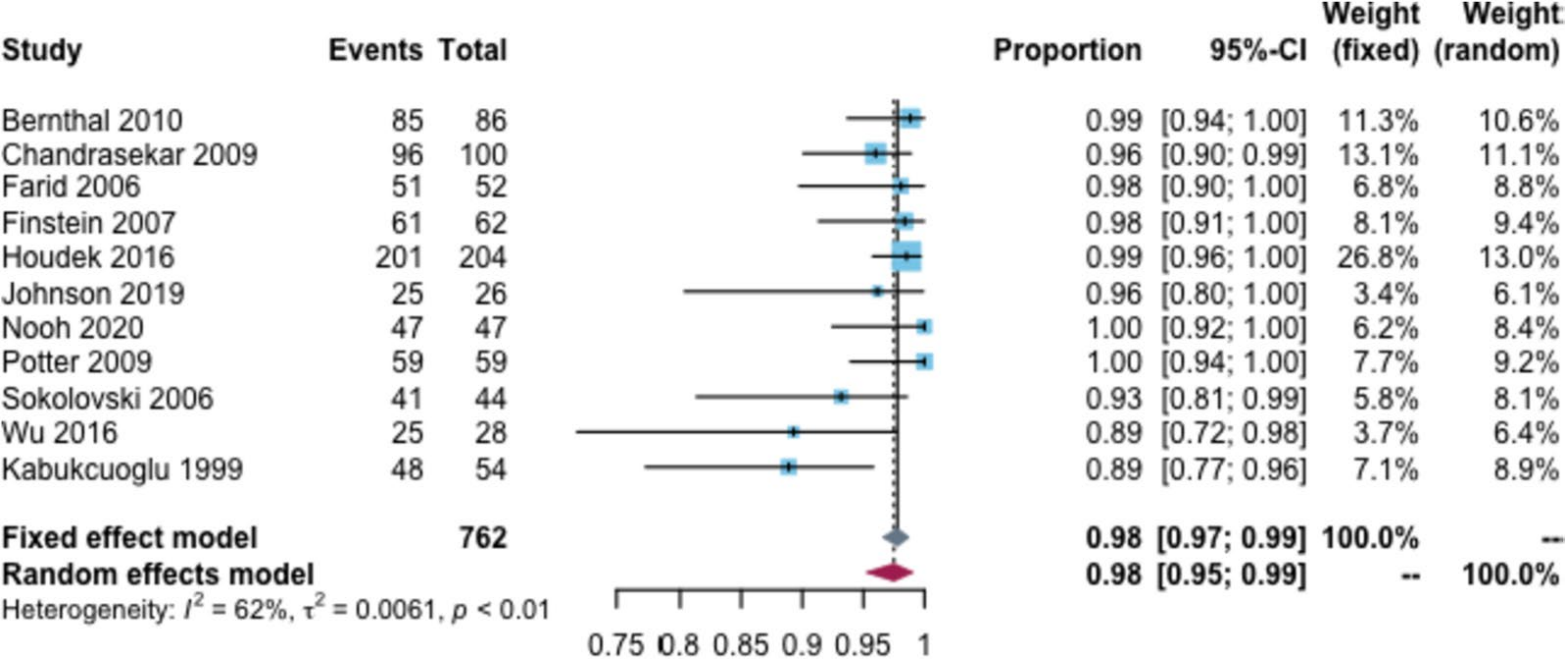
Limb salvage rate

The overall limb salvage rate was 98% (95% CI: 95–99%, I^2^ = 65%) (Fig. 3). The follow-up time subgroup analysis showed that the limb preservation rates were 98 and 97% at 2–5 and 6–10 years, respectively. The prosthesis type subgroup analysis showed that the limb salvage rate was 91% for custom-made prostheses and 99% for hemiarthroplasty (Additional file 2: Appendix Table 4).

### Local recurrence rate of tumors

**Fig. 4:**
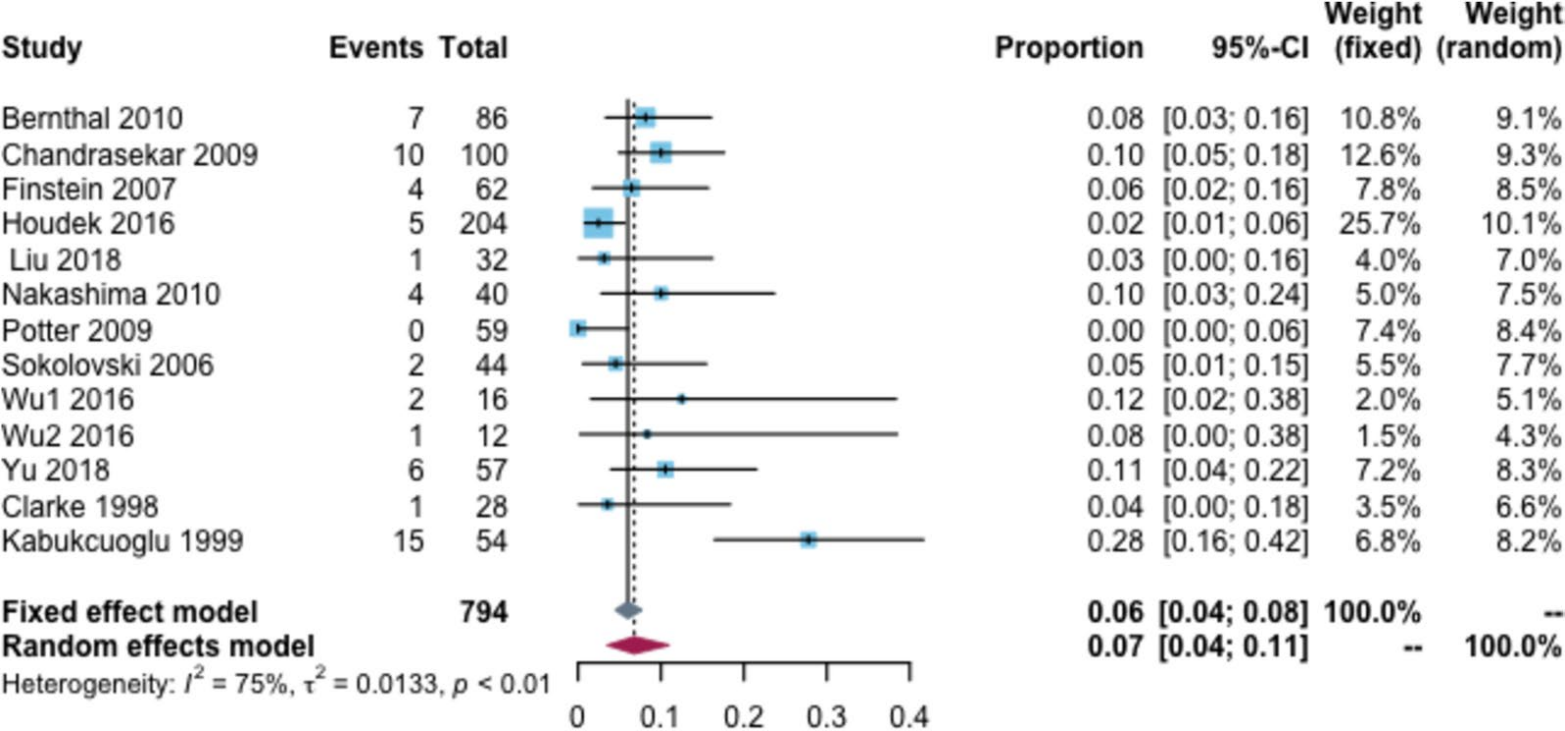
Local recurrence rate

Moreover, the local recurrence rate was 7% (95% CI: 4–11%, I^2^ = 75%) (Fig. 4). Based on the follow-up time subgroup analysis, the local recurrence rates were 6 and 8% at 2–5 and 6–10 years of follow-up, respectively. In the prosthesis type subgroup analysis, the recurrence rate was 13% for custom-made prostheses, 1% for modular-made prostheses, 4% for local hemi hip recurrence, and 5% for local recurrence of the total hip (Additional file 2: Appendix Table 5).

### Complications

Forty-six complications, such as infections, dislocations, and visceral injuries, were reported in 2069 patients from 24 cohorts. The five main complications reported were infections, dislocations, acetabular wear, deep vein thrombosis, and aseptic loosening.

**Fig. 5:**
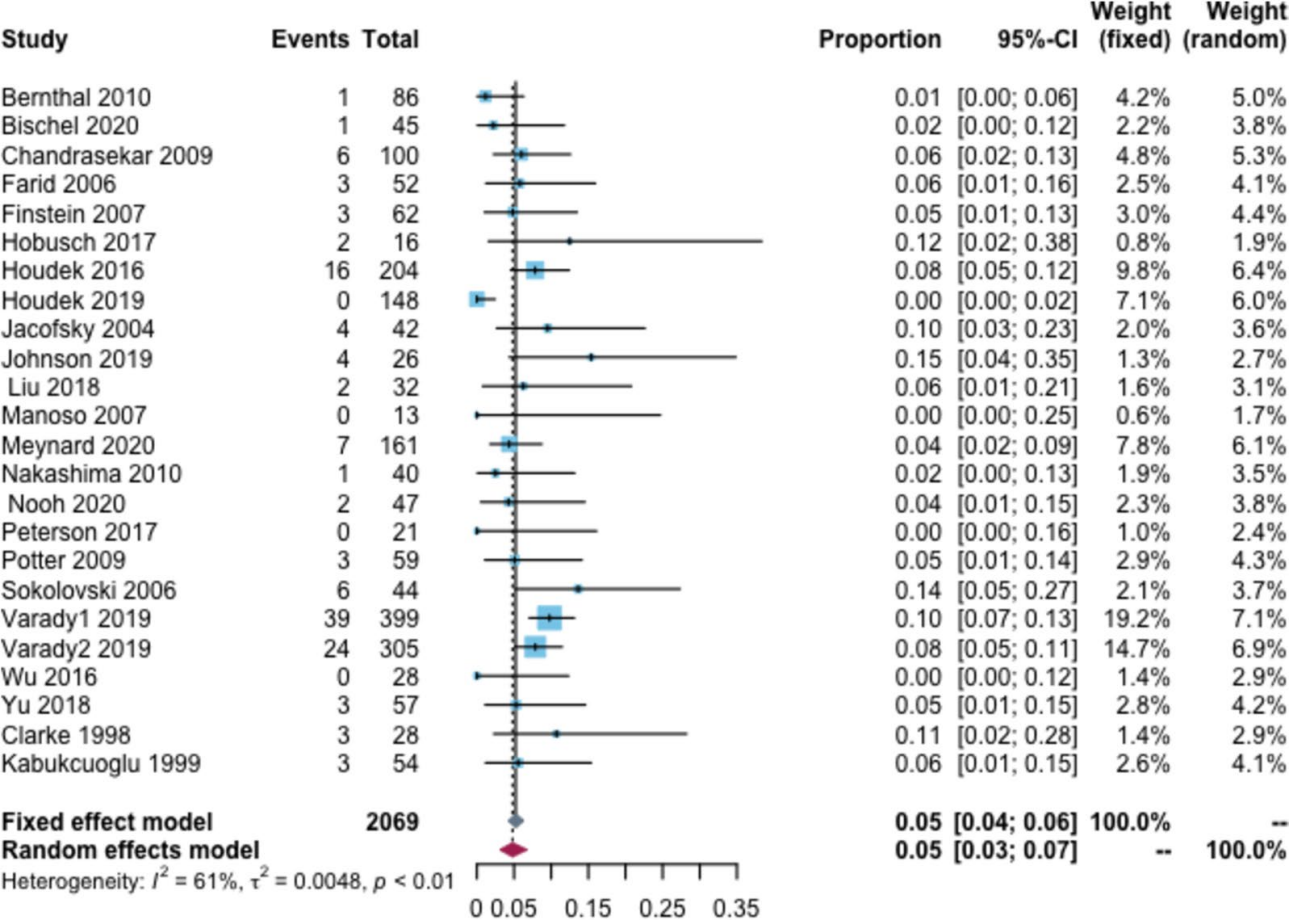
Rate of infection

Sixty-eight patients reported infections, including deep and superficial infections. The overall infection rate was 5% (95% CI: 3–7%, I^2^ = 61%) (Fig. 5). Additionally, the infection rate was 1, 3, 7, and 12% for follow-up < 2, 2–5, 6–10, and > 10 years, respectively. In the tumor type subgroup analysis, the infection rate was 5% in patients with primary tumors and 2% in those with bone metastases. In the prosthesis type subgroup analysis, the infection rate was 4% for custom-made prostheses, 3% for modular-made prostheses, 3% for hemi hip replacement, and 8% for total hip replacement (Additional file 2: Appendix Table 6).

**Fig. 6:**
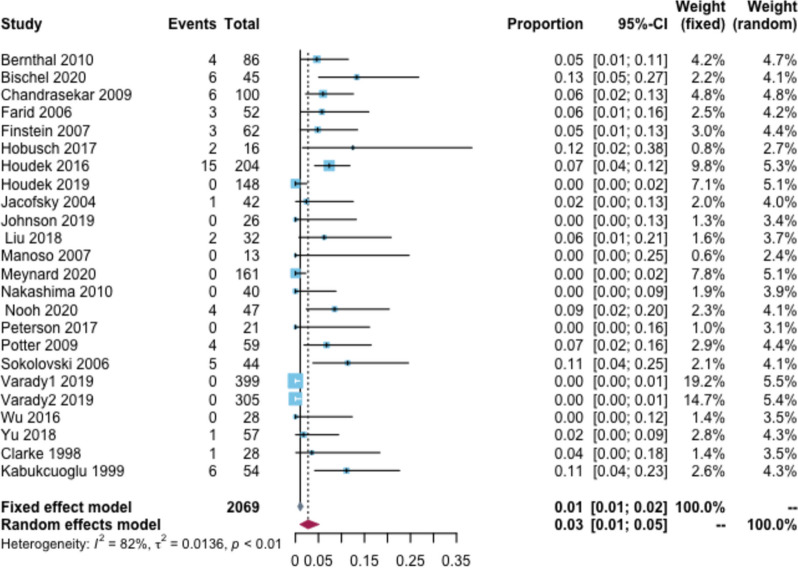
Rate of dislocation

Furthermore, 63 patients reported dislocations, with an overall rate of 3% (95% CI: 1–5%, I^2^ = 82%) (Fig. 6). Based on the follow-up time subgroup analysis, the dislocation rate at < 2, 2–5, 6–10, and > 10 years was 0, 4, 6, and 12%, respectively. In the tumor type subgroup analysis, the dislocation rate was 5% in patients with primary tumors and 3% in patients with bone metastases. According to the subgroup analysis of prosthesis type, the dislocation rate was 5% for custom-made prostheses, 3% for modular-made prostheses, 2% for hemi hip replacement, and 4% for total hip replacement (Additional file 2: Appendix Table 7).

Fifty-four patients reported acetabular wear, comprising an overall rate of 0% (95% CI: 0–2%, I^2^ = 88%) (Additional file 2: Appendix Fig. 2). The acetabular wear rate was 0, 1, 0, and 0% for follow-up < 2, 2–5, 6–10, and > 10 years follow-up, respectively. In the tumor type subgroup analysis, the acetabular wear rate was 0% in patients with primary tumors and 0% in those with bone metastases. In the prosthesis type subgroup analysis, the rate of acetabular wear was 0% for custom-made prostheses, 2% for modular-made prostheses, 4% for hemi hip replacement, and 4% for total hip replacement (Additional file 2: Appendix Table 8).

Thirty patients reported deep venous thrombosis (DVT) with an overall rate of 0% (95% CI: 0–4%, I^2^ = 90%) (Additional file 2: Appendix Fig. 3). The follow-up time subgroup analysis showed DVT rate at < 2, 2–5, 6–10, and > 10 years of 0, 1, 0, and 0%, respectively. In the tumor type subgroup analysis, the DVT rate was 0% in patients with primary tumors and 0% in those with bone metastases. The prosthesis type subgroup analysis showed a DVT rate of 0% for custom-made prostheses, 2% for modular-made prostheses, 1% for hemi hip replacement, and 0% for total hip replacement (Additional file 2: Appendix Table 9).

**Fig. 7:**
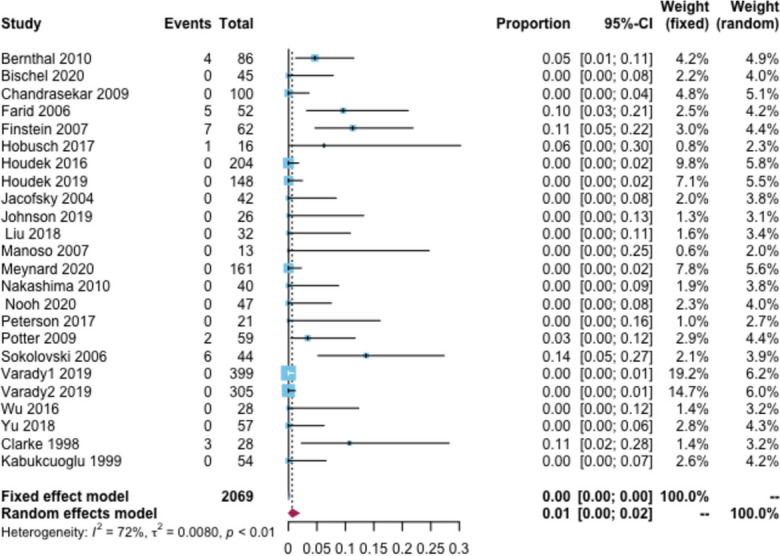
Rate of aseptic loosening

Moreover, 28 patients reported aseptic loosening, with an overall rate of 1% (95% CI:0–2%, I^2^ = 72%), Fig. 7. The follow-up time subgroup analysis showed that the aseptic loosening rate was 0, 4, 6, and 12% for < 2, 2–5, 6–10, and > 10 years, respectively. In the tumor type subgroup analysis, the aseptic loosening rate was 5% in patients with primary tumors and 3% in patients with bone metastases. The prosthesis type subgroup analysis showed that the aseptic loosening rate was 5% for custom-made prostheses, 5% for modular-made prostheses, 2% for hemi hip replacement, and 4% for total hip replacement (Additional file 2: Appendix Table 10).

### Sensitivity analysis and publication bias

The funnel plots and Egger regression presented *p* > 0.05, suggesting no publication bias. The exclusion of each study did not affect outcome indicators, indicating robust results.

## Discussion

Herein, patient clinical and prosthetic outcomes were analyzed using data from 24 studies with 2081 patients with joint prosthesis replacement after proximal femoral tumor resection. The overall patient limb preservation rate was 98%. The total prosthesis revision rate was 9% and gradually increased with increasing follow-up time. The survival rates at 1, 2, 3, 4, and 5 years were 80, 72, 65, 64, and 55% for patients with primary tumors and the rate at 1, 2, 3, 4, and 5 years were 44, 25, 17, 14, and 11% for patients with bone metastases, respectively. The rate of infection, dislocation, acetabular wear, DVT, and aseptic loosening were 5, 3, 0, 0, and 1%, respectively.

According to the tumor type classification, patients with primary tumors still had more than a 50% survival rate in the fifth year, and the trend of survival rate decreased more slowly. Meanwhile, more than 50% of patients with bone metastases had already died in the first year, and survival rate decreased by almost half in the second year. This finding was consistent with Houdek et al. [19], where the overall patient survival was associated with the presence or absence of tumor metastasis [hazard ratio (HR) 2.96; 95% CI: 2.11–4.20]. The prosthesis type analysis showed that patients with total hip replacement had a higher survival rate than those with hemi-hip replacement. The total hip replacement studies in this systematic review [30, 32] included mostly patients with primary tumors of the proximal femur or chemotherapy-sensitive proximal femoral malignancies. They have a relatively better prognosis than chemotherapy-insensitive malignant patients with higher requirements for long-term function. The total hip replacement has a better socket-prosthesis head fit, with friction occurring at the prosthesis interface rather than the prosthesis-chondral interface, and has a better long-term postoperative function. The total hip replacement patients in this study used custom prostheses, so the subgroup analysis shown that patients with custom prostheses had a higher survival rate than those with modular-made prostheses. Meanwhile, survival rates for patients with modular tumors were worse may well be because these were used in patients with metastases who were not expected to live so long considering convenience and economy.

Prosthesis survival rates were much higher than patient survival rates at the same follow-up time. This finding suggested that for patients with poor oncologic outcomes, joint prostheses can successfully preserve functional limbs without revision and for long-term survivors for at least 5–10 years. However, implant survival steadily declined over time (mean 20-year implant survival: 39%, 95% CI: 12–66%), while tumor survival declined more slowly and eventually remained stable after 10 years of follow-up. This result is similar to Liang et al. [36] regarding joint reconstruction after resectioning periprosthetic tumors in the knee.

Infection is the main cause of limb salvage failure, with deep infection being a lifelong threat after prosthetic replacement. The subgroup analysis showed a gradual increase in infection rate with increasing follow-up time, but this result should be cautiously interpreted because most studies did not provide the type of infection. The risk factors for periprosthetic infection include prolonged and repeat surgery, malnutrition, and immune compromise during comorbidities such as chemotherapy, radiation therapy, extra-articular resection, poor soft tissue coverage, hematoma formation, and diabetes mellitus [37, 38]. Various methods are available to control deep periprosthetic infections, such as amputation, level 2 revision, level 1 revision, joint replacement, irrigation, debridement, and conservative antibiotic therapy, with level 2 revision the most likely cure for infection in most infected patients [7, 39].

The hemi hip replacement has better stability than the total hip replacement. Hip dislocation was the most common complication of the upper femoral prosthesis. The subgroup analysis showed a gradual increase in dislocation rate with increasing follow-up time and a higher rate of dislocation in total hip replacements than in hemi hip replacements, similar to Thambapillary et al. [9]. Aseptic loosening is also a major complication of prosthetic reconstruction. The subgroup analysis showed a gradual increase in aseptic loosening with increasing follow-up time and a higher rate of total hip replacement aseptic loosening than hemi hip replacement. Thambapillary et al. [9] reported a 5.2% aseptic loosening, higher than the 3% reported here, which might be related to the improved prosthesis design and the choice of fixation method, making recent cases reported no aseptic loosening occurred in any of the follow-up cases [40]. Aseptic loosening is more frequent in pediatric patients than in adults [41]. Thambapillary et al. [9] included pediatric patients, while the present study included only adult patients. The pooled DVT rate was lower than the 8.5% reported by Thambapillary et al. [9]. This might be due to the emphasis on prophylactic management of post-arthroplasty thrombosis and prophylactic management of thrombosis in oncology patients in the last decade, with all study patients undergoing perioperative and postoperative prophylactic use of anticoagulants.

However, this study also has some limitations. First, due to data limitations, it was not possible to analyze the relevant outcome for specific tumor types, different prosthesis brands, resection methods, margin sizes, radiotherapy types, the combination of adjuvant radiotherapy and fixation types, and compare the impact of each factor on the outcome. Second, due to incidence limitations, the number of cases reported so far is small, and more original studies are still needed to expand the sample size of the analysis.

## Conclusion

The use of joint reconstruction after proximal femoral tumor resection to improve patients' function and quality of life has been solidified with advances in chemotherapy and radiotherapy. Proximal femoral arthroplasty has benefits in treating primary or metastatic tumors of the proximal femur. The prosthesis tends to outlive the patient, providing them with a relatively pain-free limb with good functional capacity, with limb salvage rates of 98%. Compared to total hip replacement, hemi-hip offers better stability and reduced dislocation and aseptic loosening rates.

### Supplementary Information

**Additional file 1. **

**Additional file 2: Appendix Figure 1.** Incidence of hemi to total hip conversion. **Appendix Figure 2.** Rate of acetabular wear. **Appendix Figure 3.** Rate of deep vein thrombosis. **Appendix Table 1.** Patient and prosthesis survival rates. **Appendix Table 2.** Subgroup analysis of prosthetic revision rate. **Appendix Table 3.** Subgroup analysis of hemi hip to total hip. **Appendix Table 4.** Subgroup analysis of Limb salvage rate. **Appendix Table 5.** Subgroup analysis of local recurrence rate. **Appendix Table 6.** Infection. **Appendix Table 7.** Dislocation. **Appendix Table 8.** Acetabular wear. **Appendix Table 9.** Deep Vein Thrombosis. **Appendix Table 10.** Aseptic loosening.

## Data Availability

All data generated or analyzed during this study are included in this published article.
