# Supplementary material for: Proximal femoral tumor resection followed by joint prosthesis replacement: a systematic review and meta-analysis
Source: BMC Musculoskelet Disord. 2023 Oct 2;24:779. doi: 10.1186/s12891-023-06913-w (PMC10544619; doi:10.1186/s12891-023-06913-w)
Supplement: Supplementary file 1 — Additional file 1. [file 12891_2023_6913_MOESM1_ESM.docx]

**#1 Neoplasms**

#2 Tumor

#3 Neoplasm

#4 Tumors

#5 Neoplasia

#6 Neoplasias

#7 Cancer

#8 Cancers

#9 Malignant Neoplasm

#10 Malignancy

#11 Malignancies

#12 Malignant Neoplasms

#13 Neoplasm, Malignant

#14 Neoplasms, Malignant

#15 Benign Neoplasms

#16 Benign Neoplasm

#17 Neoplasms, Benign

#18 Neoplasm, Benign

#19 OR #1-18

**#20 Arthroplasty, Replacement, Hip**

#21 Arthroplasties, Replacement, Hip

#22 Arthroplasty, Hip Replacement

#23 Hip Prosthesis Implantation

#24 Hip Prosthesis Implantations

#25 Implantation, Hip Prosthesis

#26 Prosthesis Implantation, Hip

#27 Hip Replacement Arthroplasty

#28 Replacement Arthroplasties, Hip

#29 Replacement Arthroplasty, Hip

#30 Arthroplasties, Hip Replacement

#31 Hip Replacement Arthroplasties

#32 Hip Replacement, Total

#33 Total Hip Replacement

#34 Total Hip Arthroplasty

#35 Arthroplasty, Total Hip

#36 Hip Arthroplasty, Total

#37 Total Hip Arthroplasties

#38 Replacement, Total Hip

#39 Total Hip Replacement

#40 OR #20-39

#19 AND #40
