# Supplementary material for: Proximal femoral tumor resection followed by joint prosthesis replacement: a systematic review and meta-analysis
Source: BMC Musculoskelet Disord. 2023 Oct 2;24:779. doi: 10.1186/s12891-023-06913-w (PMC10544619; doi:10.1186/s12891-023-06913-w)
Supplement: Supplementary file 2 — Additional file 2: Appendix Figure 1. Incidence of hemi to total hip conversion. Appendix Figure 2. Rate of acetabular wear. Appendix Figure 3. Rate of deep vein thrombosis. Appendix Table 1. Patient and prosthesis survival rates. Appendix Table 2. Subgroup analysis of prosthetic revision rate. Appendix Table 3. Subgroup analysis of hemi hip to total hip. Appendix Table 4. Subgroup analysis of Limb salvage rate. Appendix Table 5. Subgroup analysis of local recurrence rate. Appendix Table 6. Infection. Appendix Table 7. Dislocation. Appendix Table 8. Acetabular wear. Appendix Table 9. Deep Vein Thrombosis. Appendix Table 10. Aseptic loosening. [file 12891_2023_6913_MOESM2_ESM.docx]

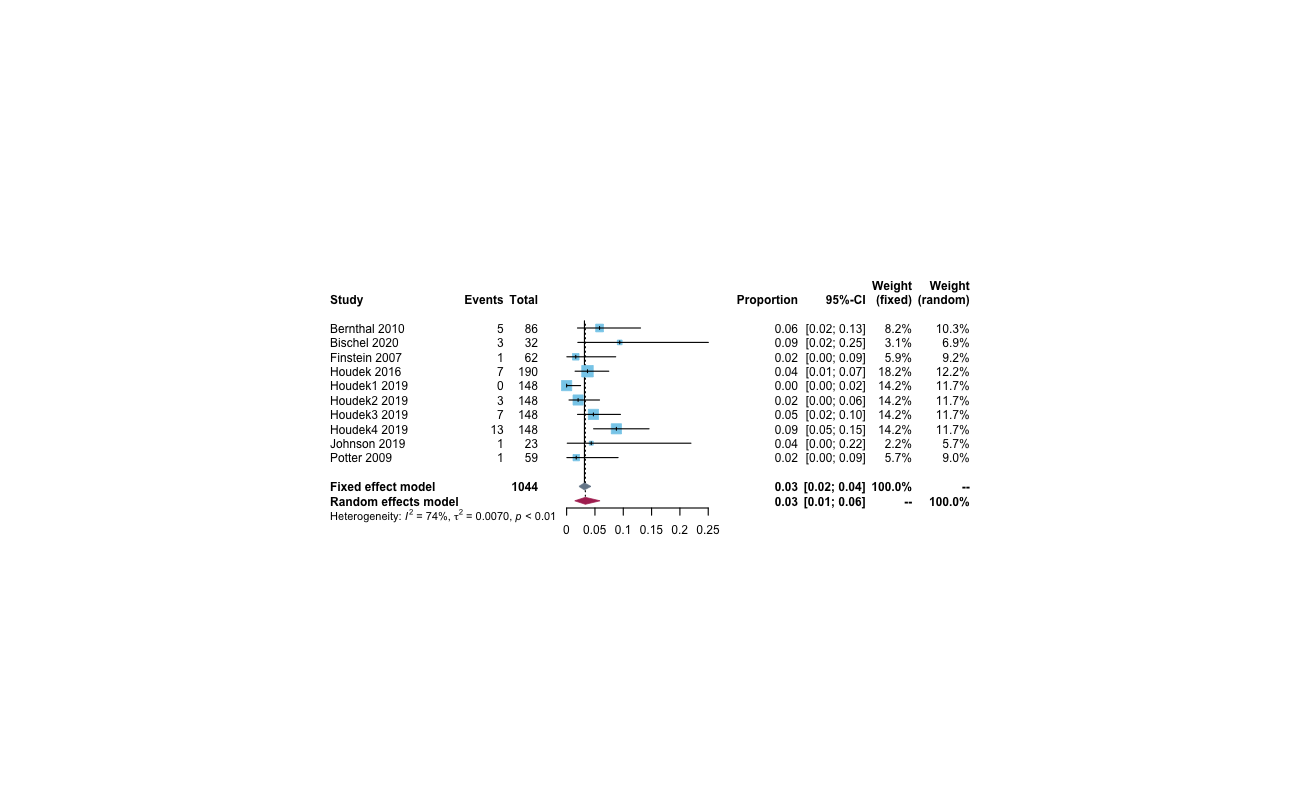


**Appendix Figure 1** Incidence of hemi to total hip conversion


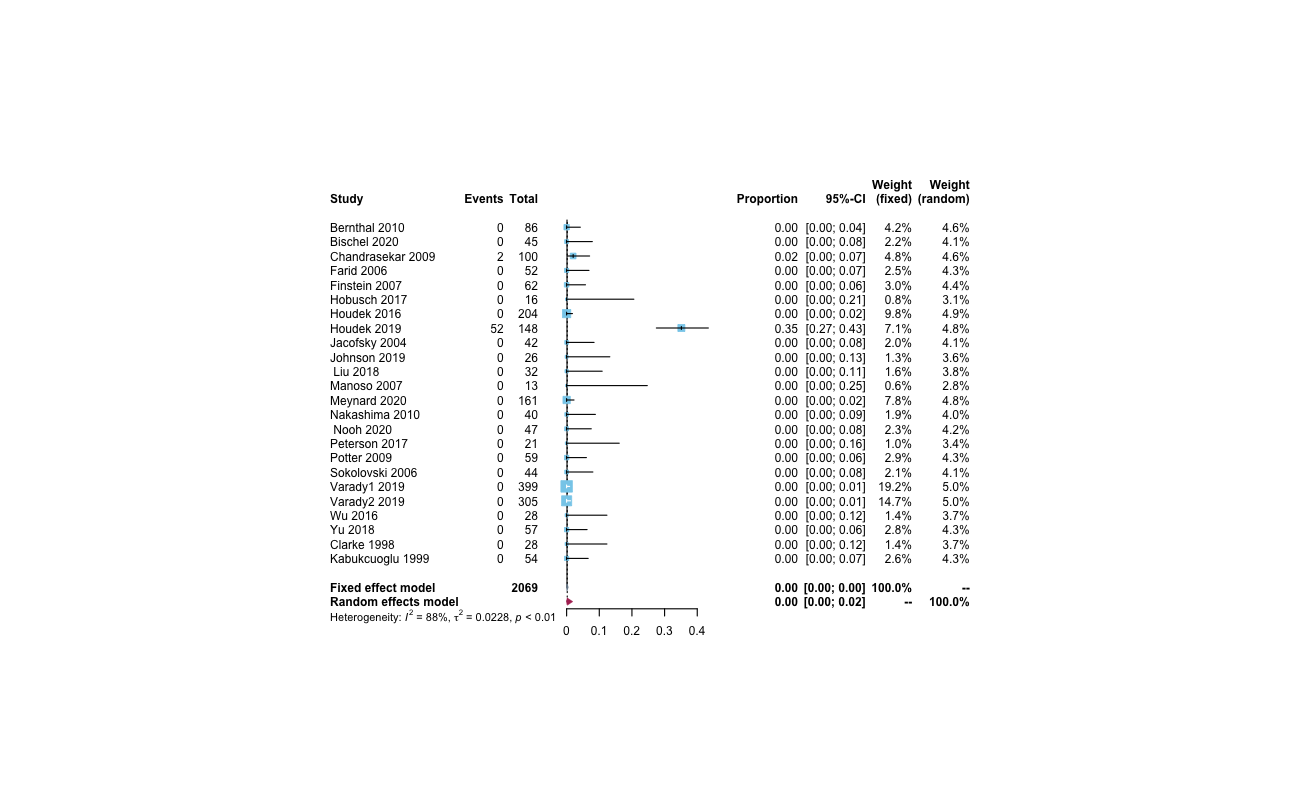


**Appendix Figure 2** Rate of acetabular wear


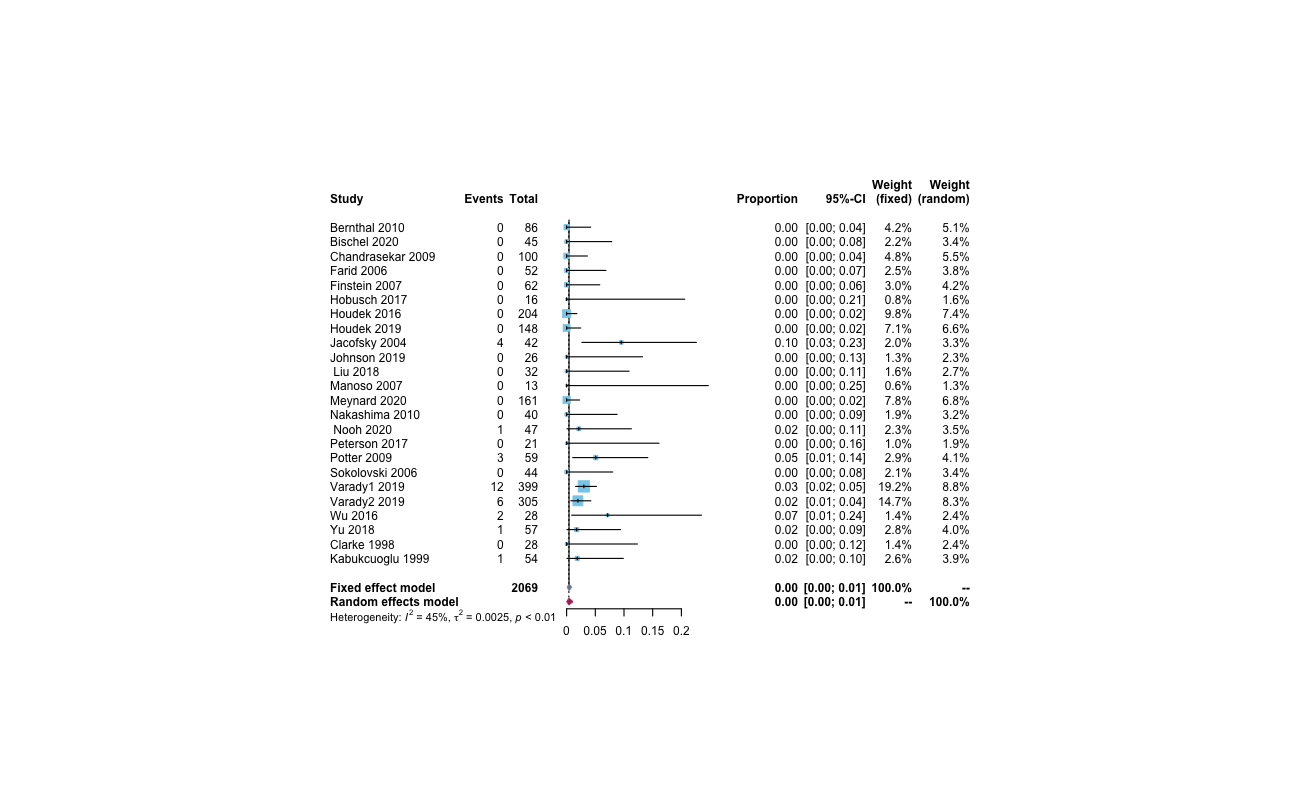


**Appendix Figure 3** Rate of deep vein thrombosis

Appendix Table 1 Patient and prosthesis survival rates

|  | Patient survival rates | | | | | Prosthesis survival rates | | | | | |
| --- | --- | --- | --- | --- | --- | --- | --- | --- | --- | --- | --- |
| Follow-up time | Study | Number of patients | rate | 95%CI | I^2^(%) | Study | Number of patients | | rate | 95%CI | I^2^(%) |
| 1^st^ year | 11 | 648 | 69% | 56-82% | 95% | 9 | 544 | 98% | | 96-100% | 56% |
| 2^nd^ year | 11 | 752 | 53% | 37-69% | 96% | 9 | 544 | 97% | | 95-100% | 60% |
| 3^rd^ year | 9 | 506 | 46% | 28-64% | 96% | 5 | 289 | 89% | | 83-96% | 72% |
| 4^th^ year | 9 | 506 | 42% | 22-62% | 98% | 5 | 289 | 88% | | 80-95% | 78% |
| 5^th^ year | 13 | 919 | 36% | 24-48% | 96% | 9 | 544 | 88% | | 80-95% | 50% |
| 10^th^ year | NA | NA | NA | NA | NA | 3 | 202 | 73% | | 58-89% | 85% |
| 15^th^ year | 4 | 437 | 28% | 12-43% | 92% | NA | NA | NA | | NA | NA |

Appendix Table 2 Subgroup analysis of prosthetic revision rate

| Subgroup | | Number of studies | Number of patients | rates | 95%CI | I^2^(%) |
| --- | --- | --- | --- | --- | --- | --- |
| Custom | 6 | | 230 | 11% | 2-28% | 91% |
| Modular | 6 | | 278 | 8% | 1-23% | 91% |
| 2^nd^ year | 4 | | 240 | 2% | 0-8% | 80% |
| 5^th^ year | 8 | | 490 | 8% | 3-14% | 75% |
| 10^th^ year | 5 | | 274 | 11% | 3-22% | 84% |
| 20^th^ year | 2 | | 49 | 47% | 33-61% | 0% |

Appendix Table 3 Subgroup analysis of hemi hip to total hip

| Subgroup | Number of studies | Number of patients | rates | 95%CI | I^2^(%) |
| --- | --- | --- | --- | --- | --- |
| Overall | 10 | 1044 | 3% | 1-6% | 74% |
| Cement | 4 | 397 | 3% | 2-5% | 0% |
| Custom | 6 | 683 | 3% | 1-7% | 84% |
| Follow up 2-5years | 4 | 387 | 2% | 0-6% | 77% |
| Follow up 6-10years | 6 | 657 | 5% | 3-7% | 25% |

Appendix Table 4 Subgroup analysis of Limb salvage rate

| Subgroup | Number of studies | Number of patients | rates | 95%CI | I^2^(%) |
| --- | --- | --- | --- | --- | --- |
| Custom | 3 | 126 | 91% | 85-95% | 0% |
| Hemi hip | 4 | 251 | 99% | 95-100% | 58% |
| Follow up 2-5years | 6 | 330 | 98% | 94-100% | 70% |
| Follow up 6-10years | 5 | 432 | 97% | 94-99% | 57% |

Appendix Table 5 Subgroup analysis of local recurrence rate

| Subgroup | Number of studies | Number of patients | rates | 95%CI | I^2^(%) |
| --- | --- | --- | --- | --- | --- |
| Custom | 4 | 126 | 13% | 3-27% | 75% |
| Modular | 2 | 91 | 1% | 0-6% | 62% |
| Hemi hip | 4 | 219 | 4% | 2-12% | 78% |
| Total hip | 3 | 92 | 5% | 2-10% | 0% |
| Follow up 2-5 years | 7 | 317 | 6% | 2-11% | 68% |
| Follow up 6-10 years | 5 | 420 | 8% | 2-16% | 86% |

Subgroup analysis of complications

Appendix Table 6 Infection

| Subgroup | Number of studies | Number of patients | rates | 95%CI | I^2^(%) |
| --- | --- | --- | --- | --- | --- |
| Primary | 3 | 110 | 5% | 1-10% | 0% |
| metastasis | 4 | 136 | 2% | 0-6% | 0% |
| Custom | 4 | 139 | 4% | 0-12% | 57% |
| Modular | 6 | 352 | 3% | 0-9% | 71% |
| Hemi hip | 7 | 820 | 3% | 0-7% | 85% |
| Total hip | 3 | 381 | 8% | 5-11% | 0% |
| Follow up <2 year | 2 | 61 | 1% | 0-7% | 0% |
| Follow up 2-5 years | 9 | 569 | 3% | 1-7% | 63% |
| Follow up 6-10 years | 6 | 488 | 7% | 4-11% | 52% |
| Follow up >10 year | 1 | 16 | 12% | 0-34% | NA |

Appendix Table 7 Dislocation

| Subgroup | Number of studies | | Number of patients | | rates | 95%CI | I^2^(%) |
| --- | --- | --- | --- | --- | --- | --- | --- |
| Primary | 3 | 110 | | 5% | | 0-19% | 76% |
| metastasis | 4 | 136 | | 3% | | 0-10% | 59% |
| Custom | 4 | 139 | | 5% | | 0-13% | 59% |
| Modular | 6 | 352 | | 5% | | 1-13% | 81% |
| Hemi hip | 7 | 820 | | 2% | | 0-7% | 86% |
| Total hip | 3 | 381 | | 4% | | 0-18% | 91% |
| Follow up <2 year | 2 | 61 | | 0% | | 0-3 | 0% |
| Follow up 2-5 years | 9 | 569 | | 4% | | 1-9% | 72% |
| Follow up 6-10 years | 7 | 488 | | 6% | | 3-9% | 23% |
| Follow up >10 year | 1 | 16 | | 12% | | 0-34% | NA |

Appendix Table 8 Acetabular wear

| Subgroup | Number of studies | | Number of patients | | rates | 95%CI | I^2^(%) |
| --- | --- | --- | --- | --- | --- | --- | --- |
| Primary | 3 | 110 | | 0% | | 0-2% | 0% |
| metastasis | 3 | 136 | | 0% | | 0-1% | 0% |
| Custom | 4 | 139 | | 0% | | 0-1% | 0% |
| Modular | 6 | 352 | | 2% | | 0-17% | 95% |
| Hemi hip | 7 | 820 | | 1% | | 0-11% | 97% |
| Total hip | 3 | 381 | | 0% | | 0-0% | 0% |
| Follow up <2 year | 2 | 61 | | 0% | | 0-3% | 0% |
| Follow up 2-5 years | 9 | 569 | | 1% | | 0-9% | 94% |
| Follow up 6-10 years | 7 | 488 | | 0% | | 0-0% | 0% |
| Follow up >10 year | 1 | 16 | | 0% | | 0-10% | NA |

Appendix Table 9 Deep Vein Thrombosis

| Subgroup | Number of studies | Number of patients | rates | 95%CI | I^2^(%) |
| --- | --- | --- | --- | --- | --- |
| Primary | 3 | 110 | 0% | 0-3% | 0% |
| metastasis | 4 | 136 | 0% | 0-2% | 0% |
| Custom | 4 | 139 | 1% | 0-5% | 13% |
| Modular | 6 | 352 | 0% | 0-2% | 31% |
| Hemi hip | 7 | 820 | 1% | 0-2% | 60% |
| Total hip | 3 | 381 | 1% | 0-2% | 0% |
| Follow up <2 year | 2 | 61 | 0% | 0-3% | 0% |
| Follow up 2-5 years | 9 | 569 | 0% | 0-2% | 43% |
| Follow up 6-10 years | 7 | 488 | 0% | 0-2% | 57% |
| Follow up >10 year | 1 | 16 | 0% | 0-10% | NA |

Appendix Table 10 Aseptic loosening

| Subgroup | Number of studies | Number of patients | rates | 95%CI | I^2^(%) |
| --- | --- | --- | --- | --- | --- |
| Primary | 3 | 110 | 5% | 0-19% | 76% |
| metastasis | 4 | 136 | 3% | 0-10% | 59% |
| Custom | 4 | 139 | 5% | 0-13% | 59% |
| Modular | 6 | 352 | 5% | 1-13% | 81% |
| Hemi hip | 7 | 820 | 2% | 0-7% | 86% |
| Total hip | 3 | 381 | 4% | 0-18% | 91% |
| Follow up <2 year | 2 | 61 | 0% | 0-3% | 0% |
| Follow up 2-5 years | 9 | 569 | 4% | 1-9% | 72% |
| Follow up 6-10 years | 7 | 488 | 6% | 3-9% | 23% |
| Follow up >10 year | 1 | 16 | 12% | 0-34% | NA |
